# The application of new complex indicators in the detection of urine

**DOI:** 10.1186/s12882-023-03087-4

**Published:** 2023-02-27

**Authors:** Ying-xiang Li, Yang Li, Si-yu Bao, Ning Xue, Xiao-qiang Ding, Yi Fang

**Affiliations:** 1 Department of Nephrology, Zhongshan Hospital, Fudan University, 111 Yixueyuan Road, Shanghai, 200032 China; 2 Shanghai Medical Center of Kidney Disease, Shanghai, 200032 China; 3 Shanghai Institute of Kidney Disease and Dialysis, Shanghai, 200032 China; 4 Shanghai Key laboratory of Kidney and Blood Purification, Shanghai, 200032 China

**Keywords:** Urinary red blood cell count, New complex indicator, Hematuria, Diagnosis, Renal pathology

## Abstract

**Background:**

Accurate diagnosis and assessment of hematuria is crucial for the early detection of chronic kidney disease(CKD). As instability of urinary RBC count (URBC) often results with clinical uncertainty, therefore new urinary indexes are demanded to improve the accuracy of diagnosis of hematuria. In this study, we aimed to investigate the benefit of applying new complex indicators based on random urine red blood cell counts confirmed in hematuric kidney diseases.

**Methods:**

All patients enrolled underwent renal biopsy, and their clinical information was collected. Urinary and blood biomedical indexes were implemented with red blood cell counts to derive complex indicators. Patients were divided into two groups (hematuria-dominant renal histologic lesions and non-hematuria-dominant renal histologic lesions) based on their renal pathological manifestations. The target index was determined by comparing the predictive capabilities of the candidate parameters for hematuric kidney diseases. Hematuria stratification was divided into four categories based on the scale of complex indicators and distributional features. The practicality of the new complex indicators was demonstrated by fitting candidate parameters to models comprising demographic information.

**Results:**

A total of 1,066 cases (678 hematuria-dominant renal histologic lesions) were included in this study, with a mean age of 44.9 ± 15 years. In differentiating hematuria-dominant renal histologic lesion from the non-hematuria-dominant renal histologic lesion, the AUC value of “The ratio of the random URBC to 24-h albumin excretion” was 0.76, higher than the standard approach of Lg (URBC) [AUC = 0.744] (95% Confidence interval (CI) 0.712 ~ 0.776). The odds ratio of hematuria-dominant renal histologic lesion (Type I) increased from Q2 (3.81, 95% CI 2.66 ~ 5.50) to Q4 (14.17, 95% CI 9.09 ~ 22.72). The predictive model, composed of stratification of new composite indexes, basic demographic characteristics, and biochemical parameters, performed best with AUC value of 0.869 (95% CI 0.856–0.905).

**Conclusion:**

The new urinary complex indicators improved the diagnostic accuracy of hematuria and may serve as a useful parameter for screening hematuric kidney diseases.

## Introduction

Hematuria is a common urinary abnormality indicative of chronic kidney disease (CKD). In the long asymptomatic early phase of CKD, urinalysis was considered a high priority for evaluating patients with suspected kidney diseases that manifest as hematuria [1]. Urine red blood cells (RBCs) count, a strong indicator of hematuria, has true variability in individual patients ranging from asymptomatic to rapidly progressive stages[2]. Fluctuations in urinary component concentration are affected by water intake, exercise, and improper urine sample collection and are associated with the instability of urinary RBC count (URBC) [3]. Although limited data are available regarding the test performance of RBCs count [4], efforts are being made to clarify urine test competence and identify candidates for enhancing the accuracy of hematuria diagnosis. In this study, we aimed to optimize the URBC based routine urinalysis, explore new urinary indexes, and subsequently improve accuracy and stability in hematuria diagnosis and early detection of CKD.

## Materials and methods

### Study participants

A total of 1,066 patients were hospitalized at the Department of Nephrology, Zhongshan Hospital, Fudan University, between August 2018 and July 2021. All patients underwent renal biopsy, and their medical records (including blood chemistry and urinalysis) were retrospectively retrieved. Inclusion criteria were age > 18 years and had not yet received renal replacement therapy. The exclusion criteria were as follows: transplant recipients, pregnant women, female patients who were menstruating, patients with active tuberculosis, malignancy, acute hemorrhage, an indwelling urinary catheter, infection, or urolithiasis. The study was approved by the Clinical Research Ethical Committee of the Zhongshan Hospital, Fudan University. Informed consent was obtained from all patients. All methods were carried out in accordance with relevant guidelines and regulations.

### Clinical data and definitions

Demographic data, including age, sex, height, weight, body mass index (BMI = weight/height^2^[kg/m^2^]), history of diabetes, and hypertension, were retrieved from electronic medical records. Laboratory data from spot urine samples included urinary specific gravity (SG), urinary RBCs, dipstick protein measurements, urinary microalbumin (µmol/L), urinary creatinine level (mg/L), and the percentage of dysmorphic RBCs (%). Quantitative measurements of urine chemistry and protein levels were also collected. Blood chemistry included serum urea nitrogen (mmol/L), estimated glomerular filtration rate (eGFR[ml/min/1.73 m^2^]), serum creatinine (µmol/L), uric acid (µmol/L), and albumin (g/L] ).

Laboratory data were collected from the latest pre-renal biopsy tests. Urinary blood cell count was performed simultaneously within 2 h after collection and was recorded as RBCs/µL using an automated method with a urine sediment analyzer (UF-1000, Sysmex). Microscopic urine examination was performed using a bright-field microscope according to the recommendations of the Clinical & Laboratory Standards Institute (CLSI) guidelines as RBC/high-power field (HPF) [5]. Microscopic hematuria was defined as three or more RBCs/high-power field [6] or > 25 RBC/µL [7] using the automated method. The percentage of dysmorphic RBCs was calculated using phase-contrast microscopy. Urine dipstick protein was categorized as negative, +, ++, or +++.

The urinary albumin-to-creatinine ratio (ACR, µg/mgCr) was calculated as the urinary albumin concentration divided by the urinary creatinine concentration, and the protein-to-creatinine ratio (PCR) was calculated as the urinary protein concentration divided by the urinary creatinine concentration. Estimated GFR (eGFR) was calculated according to the Chronic Kidney Disease Epidemiology Collaboration (CKD-EPI) Equation. [8]. Hypertension was defined as a systolic blood pressure ≥ 140 mmHg or diastolic blood pressure ≥ 90 mmHg [9]. Diabetes was defined according to the new criteria of the American Diabetes Association and provisional criteria of the World Health Organization [10].

### Renal biopsy-based pathological classification and criteria

Ultrasound-guided percutaneous renal biopsy was routinely performed at the core laboratory of Zhongshan Hospital, Fudan University. Renal pathologists collaborated with a nephrologist to make a definitive clinicopathological diagnosis. Renal pathological assessment was performed using light microscopy, immunofluorescence microscopy, and electron microscopy.

Based on the pathological lesions detected in renal biopsy combined with clinical manifestations, the spectra of kidney diseases were classified into two types to identify the correlation between RBC measurement in urinalysis and pathological diagnosis. Type I, hematuria-dominant renal histologic lesion; Type II, non-hematuria-dominant renal histologic lesion. Type I features proliferative lesions associated with hematuria and is usually characterized by diffuse mesangial cell proliferation and/or capillary proliferation with or without extensive crescent formation, often with renal interstitial inflammation [11]. Segmental fibrinoid necrosis of the glomerular tuft has been observed in severe cases. Mesangial cell proliferative lesions coexisting with segmental sclerosis and/or capsular adhesion can also be observed [12]. Mesangial cell proliferative glomerulonephritis such as IgA nephropathy [13], endocapillary proliferative glomerulonephritis, membranoproliferative glomerulonephritis, and crescentic glomerulonephritis exhibit hematuria-dominant renal histologic lesions [14]. In our study, pathological patterns presenting with proliferative lesions confirmed by biopsy were classified as Type I. Type II involves pathological patterns with nonproliferative glomerular lesions [15]. Membranous nephropathy, minimal change disease, focal segmental glomerulosclerosis (FSGS), hypertensive nephropathy, diabetic nephropathy, renal amyloidosis, and tubulointerstitial nephropathy manifest as non-proliferative lesions [16].

### Identification of new urinary complex indicators

Complex indicators that adjust the parameters for surrogates of routine urinalysis RBC counts were developed to yield hematuria measurements. The relationship between urinalysis results, biochemical indices, and renal pathological classification was analyzed. Urinary parameters included URBC, creatine level, albumin, albumin-creatinine ratio, 24-h urine protein excretion, and 24-h creatinine excretion. The serum biochemical parameters included creatinine, albumin, and urea nitrogen levels. We determined a series of new complex indicators based on the URBC, which included the ratio of random URBC to urinary specific gravity (URBC/SG), the ratio of random URBC to urinary microalbumin (URBC/UAlb), the ratio of random URBC to urinary creatinine (URBC/UCr), the ratio of random URBC to 24-h urinary protein quantification (URBC/24 hUP), and the ratio of random URBC to 24-h urinary creatinine quantification (URBC/24 hUCr).

### Hematuria stratification and model establishment

The entire range of parameters was stratified into four equal parts based on the degree of hematuria. Hematuria stratification was defined accordingly, and their correlations with hematuria-dominant renal histologic lesions (Type I) were analyzed. Three models of the new urinary complex indicator (Lg(URBC/24 hUP)), combining diverse factors, were constructed. Models adjusted for demographic characteristics, past medical history, and biochemical data were utilized separately based on hematuria stratification. The relationship between the three models and Type I was analyzed. Model 1 was a univariate model with hematuria stratification alone, Model 2 = Model 1 + demographics (age, sex, and BMI) and comorbidities (diabetes and hypertension), and Model 3 = Model 2 + biochemical data (eGFR and albumin level).

### Statistical analysis

Statistical analysis was performed using Statistical Package for Social Sciences (SPSS), version 25.0 (SPSS Inc., Chicago, IL, USA). Qualitative data were presented as numbers and percentages (%). Quantitative data were presented as means with standard deviations (SD) for continuous variables with approximately normal distributions and interquartile ranges (IQR) for non-normally distributed data. All indexes with skewed distributions were log-transformed and dimension-transformed to obtain normal distributions. An independent sample t-test was used to compare the group differences for normally distributed continuous variables. The non-parametric rank-sum test was used to compare group differences for non-normally distributed data. All categorical data were analyzed using the χ^2^ test. The differences in the distribution of each urinary index between patients with Type I and Type II were compared. The logarithm of the composite index was analyzed, and a receiver operating characteristic (ROC) curve was drawn. The predictive performance of each new urinary complex indicator for hematuria-dominant renal histological lesions was assessed by calculating the area under the ROC curve (AUC). We classified the composite index into four categories for hematuria stratification. The Cochran-Mantel-Haenszel test was used to evaluate hematuria stratification. Multivariate logistic regression models were fitted to the data to test the relationship between the new urinary complex indicators, and Type I and AUC was used to evaluate predictive capacity. Statistical significance was set at p < 0.05 for a 2-sided test.

## Results

### Clinical and demographic characteristics

A total of 1,066 hospitalized patients were included in this study. The mean age was 44.9 ± 15.0 years; men accounted for 59.1% (n = 630). Of these patients, 139 (13.0%) had a history of diabetes, and 552 (51.8%) had hypertension.

In the data obtained from the Shanghai Laboratory of Kidney Disease and Dialysis, 872 patients were diagnosed with nephritic glomerular disease (674 with IgA nephropathy, 83 with membranous nephropathy, 72 with FSGS, 34 with minimal change disease, and 9 with crescentic glomerulonephritis), 15 with lupus nephritis, 11 with Henoch-Schönlein purpura, and 3 with hepatitis B virus-related glomerulonephritis. Additionally, 25, 30, 53, and 57 patients were diagnosed with tubulointerstitial nephropathy, hypertensive nephropathy, diabetic nephropathy, and other types, respectively. Of these, 736 (69.0%) patients were classified as hematuria-dominant renal histologic lesions (Type I), and the other 330 (30.9%) patients were classified as non-hematuria-dominant renal histologic lesions (Type II). The clinical and pathological characteristics were described in Table 1.

**Table 1:** Characteristics by pathologic classification and distribution

Characteristics	Pathologic classification and distribution				
	Type I(N = 724)	Type II(N = 342)	Total (N = 1066)	Statistics	P-value
**Demographics**					
age, years	41.77 ± 13.71	51.38 ± 15.53	44.85 ± 15.00	-8.545	< 0.001^*^
male/female	399/325	231/111	630/436	14.855	< 0.001^†^
history of hypertension	350(48.3%)	202(59.1%)	552(51.8%)	10.694	0.001^†^
history of diabetes	40(5.5%)	99(28.9%)	139(13.0%)	112.381	< 0.001^†^
BMI	24.24 ± 3.88	25.05 ± 3.95	24.50 ± 3.92	-3.101	0.002^*^
**Urinalysis**					
urinary RBC(analyzer)	64.0[20.8,207.3]	13.0[3.0,42.0]	39.5[10.0,133.0]	——	< 0.001^#^
urinary RBC/HP(microscopy)	5.0[0,30.0]	0.0[0.0,4.0]	4.0[0.0,20.3]	——	< 0.001^#^
dysmorphic percentage(%)	5%[0%,50%]	0%[0%,5%]	5%[0%,14%]	——	< 0.001^#^
specific gravity	1.02 ± 0.01	1.02 ± 0.01	1.02 ± 0.01	-0.715	0.475^*^
urinary creatine(µmol/L)	9128[6292,13406]	6744[4876,10008]	8413[5748,12511]	——	< 0.001^#^
microalbumin(mg/L)	587[275,1300]	746[172,2227]	617[248,1473]	——	0.061^#^
ACR(µg/mgCr)	602[231, 1317]	888[190,2792]	634[221,1713]	——	< 0.001^#^
24-h urinary protein excretion(g/24 h)	1.14[0.65,2.12]	1.55[0.58,4.53]	1.24[0.64,2.75]	——	< 0.001^#^
24-h urinary creatinine excretion(µmol/24 h)	10,259[7971,13156]	10,000[7844,13246]	10,201[7907,13170]	——	< 0.001^#^
**Biochemical index**					
creatinine(µmol/L)	104[77,146]	110[77,174]	105[77,155]	——	0.221^#^
eGFR(EPI)(ml/min/1.73m^2^)	68.30 ± 31.87	64.18 ± 33.65	66.97 ± 32.50	-1.936	0.053^*^
albumin(g/L)	38.21 ± 5.48	34.81 ± 8.64	37.10 ± 6.86	-7.656	< 0.001^*^
urea nitrogen(mmol/L)	6.3[5.0,8.5]	7.5[5.4,10.7]	6.6[5.0,9.6]	——	< 0.001^*^

* Student’s t-test; # Mann–Whitney test; †Pearson test

BMI, body mass index; HP, high power field; GFR, glomerular filtration rate; ACR, albumin to creatinine ratio

### Distributions of indexes in pathological classification

Among urinary parameters, 24-h urinary protein excretion significantly differed between Types I and II (1.14 g/24 h vs. 1.55 g/24 h, *p* < 0.001). The 24-h urinary creatinine excretion was higher in Type I (10,259 µmol/24 h vs. 10,000 µmol/24 h, *p* < 0.001) than in Type II. As for patients’ biochemical data, no significant differences in serum creatinine, eGFR, and uric acid were observed between Types I and II kidney diseases (*p >* 0.05). Serum urea nitrogen (6.3 mmol/Lvs.7.5 mmol/L, *p* < 0.001) level was higher in Type II kidney diseases than in Type I kidney diseases. In contrast, serum albumin (38.21 ± 5.48 g/L vs. 34.81 ± 8.64 g/L, *p* < 0.001) was lower in Type II kidney diseases than in Type I kidney diseases (Table 1).

### Predictive performance of new urinary complex indicators

**Fig. 1:**
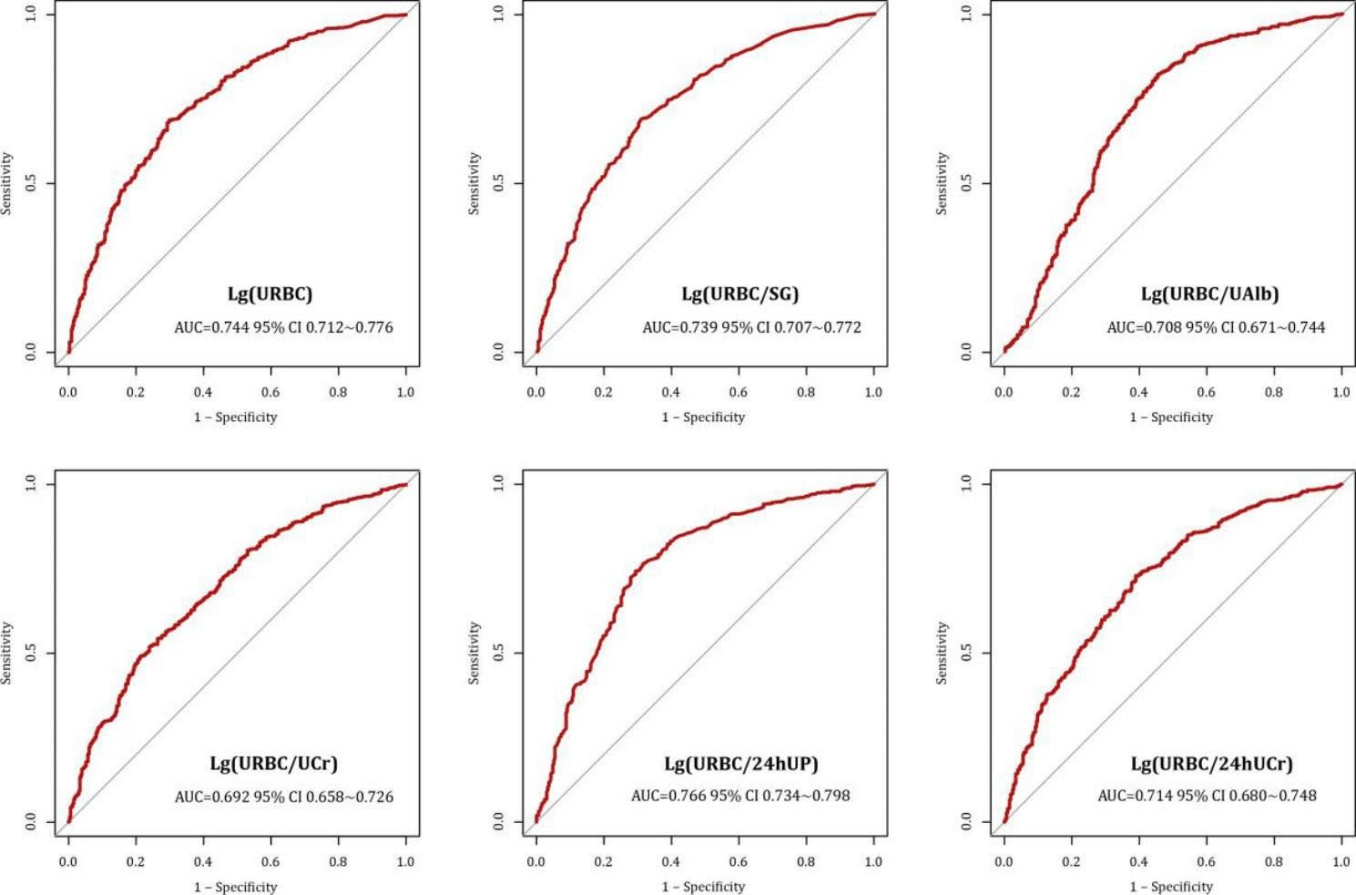
Predictive capability of new urinary complex indicators. (The predictive capability was measured by ROC-curve with AUC)

We generated five URBC-based new urinary complex indicators referring to each candidate’s correlation with Type I according to Sect. 2.2. As shown in Fig. 1, the AUC value of Lg (URBC) for predicting Type I was 0.744 (95% CI 0.712 ~ 0.776) at baseline, whereas the prediction ability of the complex indicators was ranked from high to low as follows: Lg (URBC/24 hUP) [AUC = 0.766] > Lg (URBC/SG) [AUC = 0.73] > Lg (URBC/24 hUCr) [AUC = 0.714] > Lg (URBC/UAlb) [AUC = 0.708] > Lg (URBC/UCr) [AUC = 0.692]. The Delong test confirmed that URBC/24 hUP was superior to URBC alone in predicting hematuria-dominant renal histologic lesions (Type I), with p-values of 0.002 and 0.012, respectively.

### Correlation between Lg (URBC/24 hUP) stratification and hematuria-dominant renal histologic lesions

Lg (URBC/24 hUP) was divided into quartiles characterized as Q1 (< 0.85 µL/(g/L)), Q2 (0.85 ~ 1.50 µL/(g/L)), Q3 (1.50 ~ 2.06 (µL/(g/L)) and Q4 (≥ 2.06 µL/(g/L)). The corresponding URBC/24 hUP ratios were < 7 µL/(g/L), 8 ~ 32 µL/(g/L), 33–115 µL/(g/L), and ≥ 116 µL/(g/L) (Table 2).

**Table 2:** Correlation between hematuria stratification and pathological classification

Lg(URBC/24hUP)	URBC/24hUP (µL/(g/L))	Type I N = 716(%)	Type II N = 330(%)	Odds ratio(95%CI)	P-value
Q1(< 0.85)	< 7	93(35.5)	169(64.5)	Ref(1.0)	
Q2(0.85 ~ 1.50)	8 ~ 32	174(67.7)	83(32.3)	3.81(2.66 ~ 5.50)	< 0.001
Q3(1.50 ~ 2.06)	33 ~ 115	215(81.7)	48(18.3)	8.14(5.48 ~ 12.27)	< 0.001
Q4(≥ 2.06)	≥ 116	234(88.6)	30(11.4)	14.17(9.09 ~ 22.72)	< 0.001

*20 participants were not available due to lacking 24-h urinary protein excretion;

Compared with the minimized Lg (URBC/24 hUP) level, the odds ratio of Type 1 increased from Q2 (3.81, 95% CI 2.66–5.50) to Q4 (14.17, 95% CI 9.09–22.72). There was a 3.72-fold increase in the odds ratio for doubling of Lg (URBC/24 hUP) in the range of 0.85 ~ 1.50. This indicated that a higher quartile of Lg (URBC/24 hUP) facilitated the detection of hematuria-dominant renal histological lesions with higher discrimination (Table 2).

### Performance of predictive models based on new urinary composite indexes

**Fig. 2:**
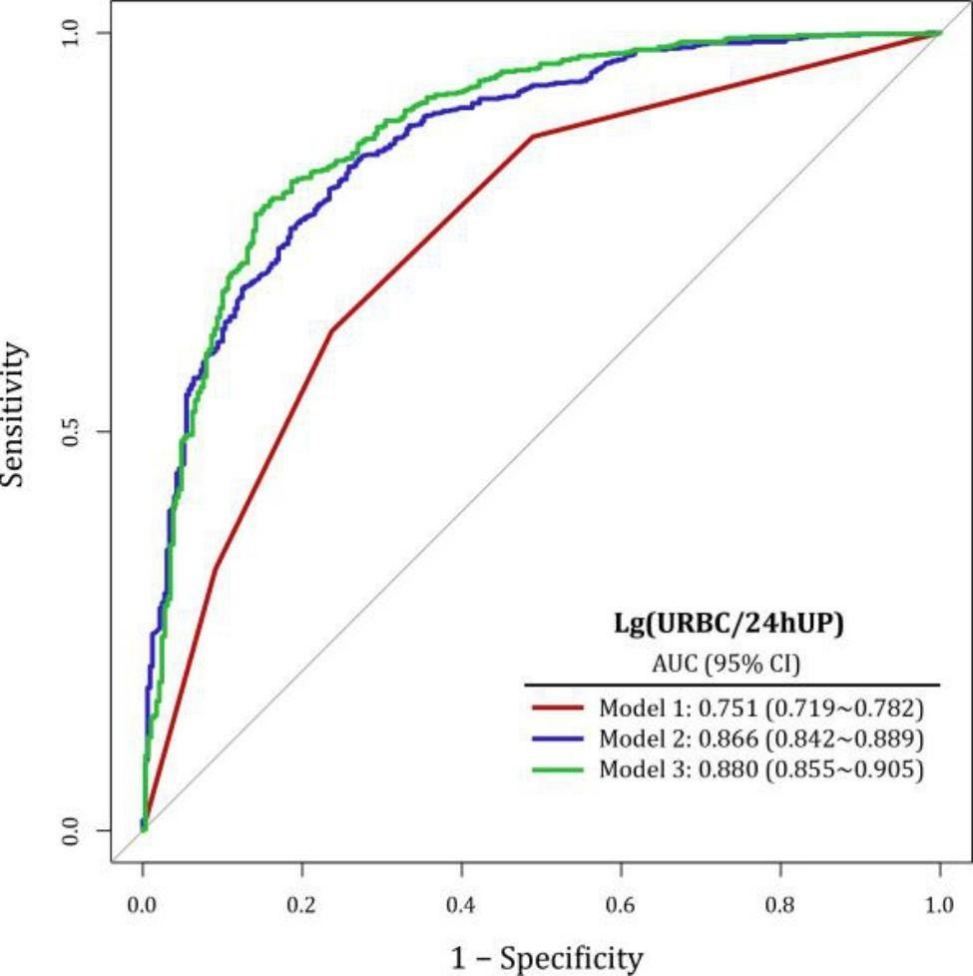
Performance of predictive models based on new urinary complex indicators. (model 1 only enrolled the Lg (URBC/24 hUP) stratification, model2 was model 1 plus demographic factors and medical history(diabetes and hypertension), model 3 was model 2 plus biochemical data)

As shown in Fig. 2, Lg (URBC/24 hUP) was applied to the three models to predict hematuria-dominant kidney disease (Type I). In Model 1, the AUC of hematuria stratification measured by complex indices for identifying hematuria-dominant renal histologic lesions (Type I) was 0.756 (95% CI 0.725–0.787). In Model 2, the AUC of the combination of hematuria stratification and demographic characteristics for identifying hematuria-dominant renal histologic lesions (Type I) was 0.869 (95% CI, 0.845–0.892). Model 3 achieved an AUC of 0.880 (95% CI, 0.856–0.905) with biochemical information.

## Discussion

Hematuria, defined as the presence of RBCs in urine, was identified by urinalysis of a concentrated urine sediment specimen [17]. Prompt referral to a nephrologist is indicated when hematuria does not resolve within weeks of onset. Effective and accurate URBC-based hematuria measurements provide explainable insights into hematuria resolution and chronic disease management. Our study investigated new complex indicators to improve URBC confirmed by a particular renal biopsy. Urine RBC counts can be measured using automated urine sediment analyzers [4]. A previous study suggested that the determination of urinary RBC distribution with an automated analyzer and analysis of the distribution curves may be a more reliable measure of erythrocyte morphology [18]. Renal biopsy, the gold standard for the diagnosis of kidney diseases, can be used to verify the sources of urinary RBCs, and the count and morphology of urinary RBCs can, in turn, provide important information on patients’ renal pathological changes [19]. In our study, we focused on hematuria originated from kidney diseases.

Furthermore, because the concentration of urine components varies greatly under the influence of drinking water, exercise, and other factors, random urine sample tests lack stability. Our findings suggest that patients with biopsy-proven non-hematuria-dominant renal histologic lesions may have high URBCs on automated urine sediment analyzers. In addition, we observed that some patients with hematuria-dominant renal histologic lesions had negative hematuria on routine urinalysis with less than 25 RBCs/µL. According to Yang et al. [20], the urine SG affects RBCs’ morphology, thus altering the identification of RBCs via automated urine sediment analyzers [21]. Considering the mechanism by which the urine sediment analyzer operates, the counting and classification of urinary RBCs are based on signals of forwarding scattered light, side scattered light, and side-fluorescent light patterns. The pattern of individual light signals is transformed by specific algorithms into individual “fingerprints,” allowing the counting, identification, and classification of the particles [22]. This inconsistency might mainly be attributed to a variable mixture of components in the urine, such as cells, casts, crystals, and bacteria. Furthermore, when the automated urine sediment analyzer interferes with various particle sizes, it may mistake other urinary components for RBCs [23]. In addition, the urine sediment analyzer itself cannot calibrate the urinary concentration or reduced kidney function. Morphological changes in urinary RBCs may be related to counting errors. Urine RBCs in a hypotonic environment exhibit a certain degree of swelling due to deteriorating kidney function [24]. We agree that URBC-based diagnosis of hematuria has certain limitations because RBC morphology and identification are unstable, with marked variability in size and shape affected by hydration status and hemodynamic status [3]. Reliable quality of URBC is critical for prognosis judgment and treatment, indicating a need for differential diagnosis [25].

We developed a parameter (URBC/24 hUP) that adjusts 24-h urinary protein quantification for surrogates of random urine RBC count to better reflect hematuria. From our study results, 24-h urinary protein quantification displayed significant distribution discrepancies in the two pathological types and was more related to Type I. Clinical practice guidelines recommend screening for and monitoring albuminuria and incorporating increased albuminuria into the definition and staging of CKD [26]. The 24-h urinary protein quantification known as the “gold standard”[27] method for evaluating proteinuria is an ideal target to be adjusted, as it avoids protein fluctuations during the day and can indirectly reflect patients’ kidney functions. The findings of “patients in the hematuria group often had overt proteinuria [11]” support the application of the 24-h urinary protein quantification.

In capturing trends in upgrading hematuria stratification-derived AUC values, we observed that patients with a higher URBC/24 hUP showed more specific interactions with Type I changes. Stratification of Lg (URBC/24 hUP) achieved an AUC value of 0.751 (95% CI, 0.719–0.782). When fitted into the model incorporating demographic characteristics and past medical history, the AUC value increased to 0.866 (95% CI 0.842–0.889). After the integration of biochemical data, the AUC further increased to 0.880 (95% CI, 0.855–0.905). These findings indicate that new urinary complex indicators corresponded better with Type I in the population at high risk of CKD, such as patients with hypertension and diabetes. For patients in the high-risk group (Q4), 24-h urine collection is a typical method of disease assessment and management. Compared to routine RBCs count based on a single collection, URBC/24 hUP corrects the urine RBC count by “24-h urinary protein quantification,” reflecting a time-accumlation effect.

Patients presenting with proteinuria and /or hematuria are usually recommended to have renal biopsy, especially other less invasive procedures are not conclusive enough[28]. Since renal biopsy is not routinely performed in remote areas, let alone inaccessibility of microscopic examinations. These non-invasive urinary complex indicators which based solely on urinalysis, will be helpful for patients in those remote areas. On the other hand, patients with contraindications of renal biopsy may also benefit from our new complex index for non-invasive diagnosis of hematuria. However, not all positive URBC results reflect factual bleeding in the urinary system due to physical and/or chemical interference and limitations of urinalysis. The correspondence of hematuria severity with values of our urinary indicator contributes to brief judgement of illness, and accelerates patients’ orientation to nephrology.

This study had some limitations. First, due to the diversity of renal pathological changes, glomeruli, renal tubules, or interstitium lesions may exist alone or together. Therefore, the corresponding urine abnormalities may interfere with one another. Second, the limited sample size and the unbalanced constituent ratio between Type I and Type II barriers further generalization. Third, a discrepancy between the urinalysis and clinical pathology manifestation existed in some cases, which might be attributed to intermittent onset of hematuria, while other patients who presented with recurrent hematuria and no urinary abnormalities between the hematuric bouts may be recorded in this study as well. As a result, patients with proliferative glomerulonephritis may have negative hematuria, especially in well-rested conditions. Failure of patients to comply with the standards of urine collection or any mistakes in urine sample transport and storage would lead to pre-analytical errors and inaccurate results [29]. However, the accuracy of urine tests can be improved by repeated urine tests. Fourth, random urine data collected at a single time point cannot fully and accurately reflect the state of kidney diseases. Last but not the least, the complex indicators were analyzed by urine RBCs intead of RBCs/HPF. The reason was that microscopy of urine RBCs is performed only when the occult (dry chemical analysis) is inconsistent with urine RBCs count (flow cytometry) in our center. Further study can be conducted to explore other complex indicators based on RBCs/HPF and extend the practical utility to centers without automated analyzers.

## Conclusion

In combination with clinical information, the accuracy of urine RBC counting could be improved, and the integrity of hematuria diagnosis could also be promoted simultaneously to achieve or approach the non-invasive diagnosis of CKD [2]. This small sample size study might provide crucial support for the diagnosis and treatment of patients who are contraindicated for renal biopsy and likely guide and support clinical activities for primary health care institutes that do not qualify to perform renal biopsy.

## Data Availability

The datasets used and/or analysed during the current study are available from the corresponding author on reasonable request.
